# Flood inundation mapping- Kerala 2018; Harnessing the power of SAR, automatic threshold detection method and Google Earth Engine

**DOI:** 10.1371/journal.pone.0237324

**Published:** 2020-08-19

**Authors:** Varun Tiwari, Vinay Kumar, Mir Abdul Matin, Amrit Thapa, Walter Lee Ellenburg, Nishikant Gupta, Sunil Thapa

**Affiliations:** 1 International Centre for Integrated Mountain Development, Dehradun, India; 2 Indian Institute of Remote Sensing, Indian Space Research Organization, Dehradun, India; 3 Earth System Science Center, University of Alabama, Huntsville, Alabama, United States of America; Bristol University/Remote Sensing Solutions Inc., UNITED STATES

## Abstract

Flood inundation maps provide valuable information towards flood risk preparedness, management, communication, response, and mitigation at the time of disaster, and can be developed by harnessing the power of satellite imagery. In the present study, Sentinel-1 Synthetic Aperture RADAR (SAR) data and Otsu method were utilized to map flood inundation areas. Google Earth Engine (GEE) was used for implementing Otsu algorithm and processing Sentinel—1 SAR data. The results were assessed by (i) calculating a confusion matrix; (ii) comparing the submerge water areas of flooded (Aug 2018), non-flooded (Jan 2018) and previous year’s flooded season (Aug 2016, Aug 2017), and (iii) analyzing historical rainfall patterns to understand the flood event. The overall accuracy for the Sentinel-1 SAR flood inundation maps of 9th and 21st August 2018 was observed as 94.3% and 94.1% respectively. The submerged area (region under water) classified significant flooding as compared to the non-flooded (January 2018) and previous year’s same season (August 2015–2017) classified outputs. Summing up, observations from Sentinel-1 SAR data using Otsu algorithm in GEE can act as a powerful tool for mapping flood inundation areas at the time of disaster, and enhance existing efforts towards saving lives and livelihoods of communities, and safeguarding infrastructure and businesses.

## Introduction

Any populated areas which normally fall in tropical regions are prone to floods which is considered as one of the hazardous natural disasters. Heavy rainfall enhances the accumulation of water in the catchments and overflow of water flashing beyond its normal confines are termed as floods. Flood usually varies from place to place and depends on numerous factors such as its severity and time of occurrence. Water inundation due to this disastrous event causes significant damage to human lives, properties, agricultural lands and infrastructure, subsequently affecting the economy [1]. Flood mitigation planning and management requires knowledge of land use as well as accurate identification and mapping of flood prone areas. Critical and timely estimation for monitoring inundated areas during floods can play a significant role in effective and prompt response for risk assessment. This also provides valuable information to the policy cum decision makers for sustainable management and future preparedness [2]. Generation of real/near real time accurate water inundation maps are required to see the flow of flood water in the affected areas during the time of disaster. Monitoring the flood extent with the help of space based sensors are more effective in providing near real time information as compared to the ground based techniques.

Space based sensors either optical or synthetic aperture radar (SAR) have shown their potential for monitoring and mapping flood or water inundated areas [3,4]. SAR sensors can penetrate through clouds and heavy rain, thus makes them capable of acquiring data during floods while optical sensors such as multispectral are unable to do the same [5]. Earlier studies have shown that SAR datasets are preferably being used for mapping and monitoring the flood extent. [6–11]. The techniques used for flood mapping are broadly categorized as (i) conventional and machine learning based classification approach [12–14]; and (ii) threshold based approach [15,16]. The first approach requires ample training data from the field which is difficult to collect during or at the time of floods. Hence, these approaches are least recommended for near real time preparation of flood maps [17,18]. However, threshold based techniques (manual as well as automatic) seem to be more effective for flood map generation. Manual threshold detection techniques are often difficult and challenging, as manually deriving the threshold requires knowledge of a few number of training samples [19–21]. This technique also leads to generation of less-accurate and inconsistent water inundated maps. This happens due to variability in the manually selected threshold values for separating water and non-water pixels [16].

However, an automatic threshold based approach detects the threshold values automatically for separating water and non-water pixels without the use of any training samples [22,23]. This approach computes image statistics for deriving the optimum thresholds to classify water and non-water pixels. Image tiling and split selection, Kittler and Illingworth’s technique, Global minimum threshold method, Quality index thresholds, Split combination and Otsu image threshold are some of the well-known automatic threshold based techniques used for different applications [24–27]. The Otsu image threshold method is the most widely adopted approach used for flood mapping [23,28,29]. This method is based on an adaptive threshold method which measures to evaluate the between-class variance of a threshold at a given level computed from a normalized image histogram. This method is less complex and requires least user interaction making it suitable for large SAR image scenes. Tests of this Otsu algorithm were conducted on eight large scenes of Radarsat-2 data for near-real time detection of water bodies and monitoring their dynamic changes [29]. An operational and robust framework was proposed for precise flood mapping by conducting tests in the large SAR scenes, for classifying flood and non-flood areas. This Otsu method is capable of providing a more precise flood map using Sentinel-1 SAR scenes for large areas [23]. Monitoring real time water extent during large-scale flood events also requires data from several sources (social media, remote sensing and topographic data). Rapid estimation of flood extent based on the information shared in real-time using social media such as geo-tagged photographs helps in validation of inundation for flood extent [30].

Various remote sensing based models with field observation have been used for monitoring and mapping of Kerala 2018 extreme flood events. Hunt et al., explored the circumstances which majorly caused this extreme flood by using Weather Research and Forecasting (WRF) model in combination with a hydrological model (WRF-Hydro, run at 125 m resolution) [31]. Haider et al., used downscaled future and historic climate projections from Coupled Model Inter comparison Project (CMIP5) and the Noah-MP land surface hydrological model, to simulate and observe the projected changes in extreme precipitation and flood events [32]. The extreme precipitation and runoff conditions observations made by Mishra et al. using the Variable Infiltration Capacity (VIC) model, have shown that the 2018 extreme flood event was unprecedented in the last seven decades from 1951–2018 [33]. The simulation results by Viswanadhapalli et al., suggested that the high convective instability and transport of mid-tropospheric moisture from the Bay of Bengal were the driving factors behind the heavy rainfall over Kerala in 2018 [34]. The change in water levels of reservoir storage during extreme rainfall of Kerala in 2018 were also discussed by Ramasamy et al., Anandalekshmi et al.,. The existing six major reservoirs that serve the state would have needed to have 34% more capacity to handle the heavy rainfall, and controlled release might have helped in alleviating the flood disasters [35,36]. These studies suggested that extreme heavy rainfall was the major factor behind this disastrous event and projected climate change could intensify these effects in the near future.

Most of the studies on Kerala flood focused on a hydrological modeling-based approach to study and analyze the reasons behind the flood event; whereas limited focus was on generating near real time water inundation maps. The hydrological model based approach are not suitable as it requires an up-to-date and accurate DEM, AWS (Automatic weather station) data as well as computing infrastructure which is often not available. Lal et al., Vishnu et al., and SK et al., utilized SAR data (Sentinel-1 & ALOS-2 PALSAR) along with a manual threshold-based, conventional and machine learning based classification approaches to prepare flood extent maps and damage assessment [37–39]. Though these studies have shown good results, they used the approach for map generation which requires ample training sample points. Also, the entire process was performed on the desktop based data processing tools which requires enormous time for downloading and processing data. This limits the timely response for providing the flood maps to different decision making agencies for immediate and adequate action. Considering this above methods in a desktop based environment are not suitable for providing rapid processing services critical for a flood response. On the other hand the cloud-based image processing platform utilizing automatic threshold methods are the possible solution. Google Earth Engine (GEE) is a cloud-based image processing platform enabling the rapid processing of such big datasets covering a large area. The GEE has publically made available numerous satellite image collections and provides image analysis functionality at large spatial scales [40]. In the existing approaches for flood mapping in Kerala no study has been performed which combines openly accessible SAR (Sentinel-1) data and automatic threshold detection techniques in a cloud computing platform (GEE).

The main objective of this study was to evaluate the potential of automatic threshold detection techniques using large SAR scenes in cloud computing platform for rapid and precise mapping of flood affected areas in Kerala, India. In this study, time-series Sentinel-1 SAR data of pre and post flood events and Otsu algorithm [41,42] were utilized in GEE for mapping flood extent or water inundated areas. Rainfall time series analysis was also performed to understand the long term rainfall trend over Kerala.

## Materials and methods

**Fig 1:**
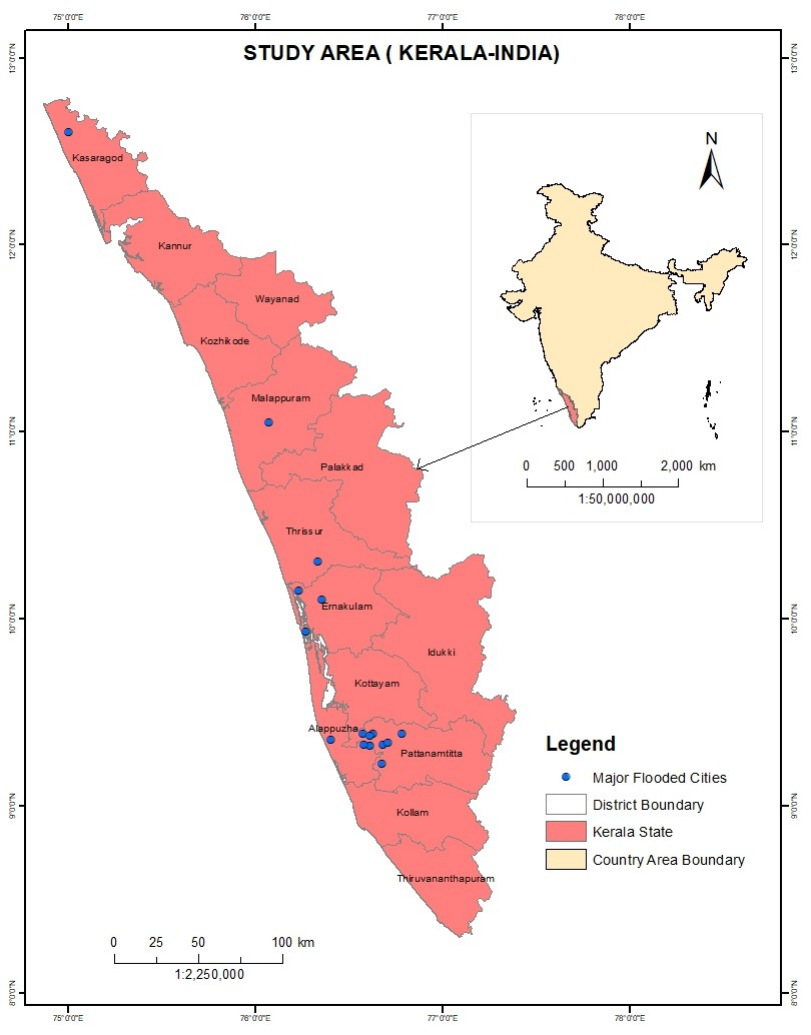
Maps of study area (Kerala India). Shapefile reprinted from GADM database under a CC BY license, with permission from Global Administrative Areas (www.gadm.org), original copyright 2018. The figure was made with ArcGIS 10.3 under a CC BY license, with permission from ESRI (www.esri.com).

The study area selected was the state of Kerala (Fig 1), situated in the southwest part of India (10.8505° N, 76.2711° E with elevation of 54 m Mean Sea Level). The area was severely affected by floods in July-August 2018 which caused substantial loss of lives, and property worth USD 5.8 billion [43]. The major cities affected were: Chengannur, Pandanad, Edanad, Aranmula, Kozhencherry, Ayiroor, Ranni, Pandalam, Kuttanad, Malappuram, Aluva, Chalakudy, Thiruvalla, Eraviperoor, Vallamkulam, N. Paravur and Cochin.

The satellite dataset utilized in the study was Sentinel 1 SAR Vertically transmitted Vertically receive (VV) Polarization data (Table 1). VV was preferred over vertically transmitted Horizontally receive (VH), as VV polarization was acquired with medium incident angle and previous research have shown it being most suitable for flood inundation mapping [44–47]. Sentinel-2 optical data and Climate Hazards Group Infra-Red Precipitation with Station data (CHIRPS) rainfall data of the flooded season (refer Table 1) were also utilized for validation of the results.

**Table 1:** Data specification.

Characteristics	Sentinel -1 (SAR data)			Sentinel– 2 (Optical data)		CHIRPS
Acquisition Date	Before Flood	Aug. 13	2015			1981–2018
		Aug. 7	2016			
		Aug. 14	2017			
		Jan. 5	2018			
		April 11				
	Starting of Flood	July 28				
	During Flood	Aug. 9 and Aug.21		Aug. 10 and Aug. 20	2018	
	After Flood	Sept. 2, Sept.16 & Sept. 26		B8-NIR		
				B4- RED		
				B3- Green		
Polarization	VV polarization			B8 –NIR; B4- RED; B3- Green		NA
Bands	1			12		NA
Wavelength	5.5cm			443-1290nm		NA
Spatial resolution	10 m			10m (resampled)		5 Km
Swath	250 Km			290 Km		NA
Temporal resolution	12 Days			5.5 Days		Daily

## Methodology

**Fig 2:**
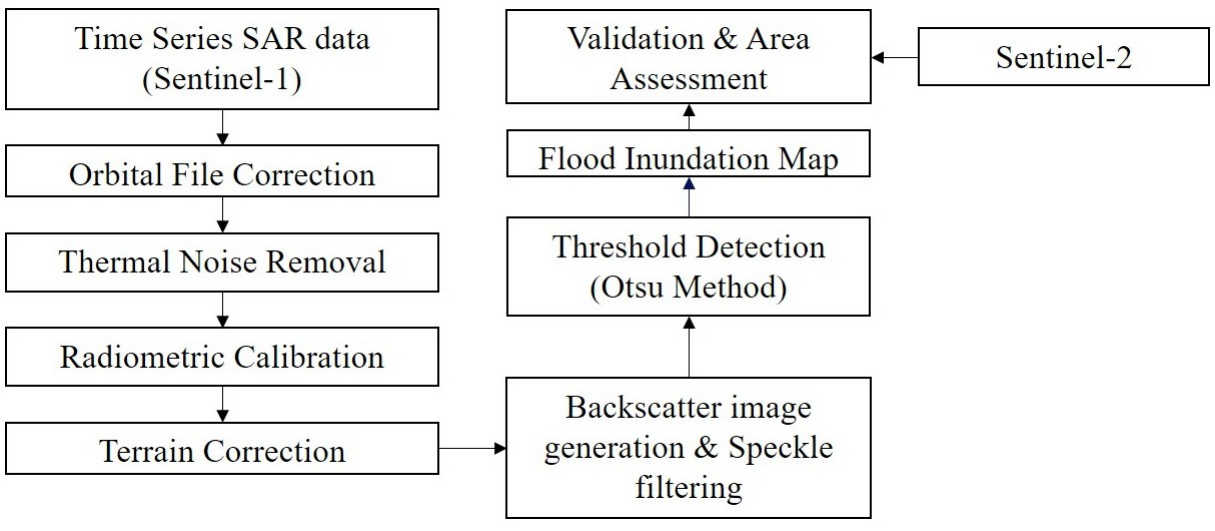
Research methodology.

The flow chart of the detailed methodology adopted in the present study is shown in Fig 2. The methodology was divided into the following steps:

### Preprocessing of Sentinel 1 VV SAR images

Sentinel-1 images suffer from erroneous noise, (i.e. rigorous geometric, radiometric corrections, thermal, and speckle) [48]. Any satellite data requires rigorous pre-processing before it can be utilized for any application.

GEE provides Sentinel-1 VV polarization Ground Range Detected (GRD) images which are already preprocessed using Sentinel-1 algorithm [48]. The preprocessing includes (i) Orbital file correction to eliminate the orbital noise; (ii) Thermal noise correction to eliminate the noise in the data produced by the sensors during data acquisition process onboard the satellite [49]. Thermal noise can affect the quality of the data in the areas having low mean signal response detected by the SAR system like lakes, standing water, rivers, etc., (iii) Radiometric calibration, to calibrate RADAR reflectivity (DN) to backscattering coefficient (physical units) which is mainly performed to compare the SAR images of different acquisition dates; and (iv) Terrain correction, to convert Sentinel-1 SLC (Single look complex) data from slant range geometry to a map coordinate system, and to rectify the distortions like foreshortening, layover, or shadowing effects. [50].

The Lee speckle filter with kernel window size 5x5 was applied in GEE environment to remove speckle noise from the dataset. [14,51]

### Classification of water and non-water areas

After preprocessing of Sentinel-1 VV SAR images, the data was classified into two major classes: water and non-water, to determine the water inundated areas. Otsu’s automatic threshold detection method was used to determine optimum threshold for this separation. The Otsu method is typically used for deriving threshold for optical data [52]. However, some recent studies have suggested that the Otsu method can also be applied in SAR data for separating water and non-water classes [53,54].

The Otsu’s method is a clustering based thresholding method used for separating two classes i.e. foreground from background. The technique assumes that the distribution of image pixel intensities follows a bi-modal histogram, and separates those pixels into two classes (e.g. foreground and background). The optimum threshold value is determined by minimizing the weighted sum of within-class (intra-class) variances of the foreground and background pixels. The mathematical algorithm of Otsu method is described as follows:

The pixels in a given image be represented in L gray levels (1, 2, 3, L). *n*_*i*_ Represents the number of pixels at level *i*, and *N* denote the total number of pixels,

$$N=n_{1}+n_{2}n_{3}+n_{L}$$ (1)

The normalized grey level histogram and probability distribution can be expressed as Eqs 2 to 4

$$P_{i}=n_{i}/N$$ (2)

$$P_{i}\geq 0$$ (3)

$$\sum_{i=1}^{L}P_{1}=1$$ (4)

Threshold k divides the pixels into two classes (i.e. foreground and background). The optimum value of k can be computed by maximizing the intra class variance (within the class). The criterion function ρ is introduced and defined as

$$\rho (k)=\sigma_{B}^{2}(k)/\sigma_{BT}^{2}$$ (5)

$\sigma_{B\;}^{2}$ and $\sigma_{BT\;}^{2}$ can be expressed as:

$$\sigma_{B}^{2}=\omega_{0}\;\omega_{1}{\left(\mu_{1}-\mu_{0}\right)}^{2}$$ (6)

$$\sigma_{T}^{2}=\sum_{i=1}^{L}{\left(i-\mu_{T}\right)}^{2}\eta_{i}$$ (7)

Where,

*ρ* = criteria function

$\sigma_{B}^{2}$ = Between class variance

$\sigma_{BT}^{2}$ = Total variance

*ω*_0_ = Probabilities of class occurrence for background

*ω*_1_ = Probabilities of class occurrence for foreground

μ_*0*_ = Class mean of background

μ_1_ = Class mean of foreground

μ_*T*_ = Total mean grey level of the image

k = Threshold value

Eqs 8 and 9 defines the probabilities of class occurrence and the class mean levels.

$$\omega (k)=\sum_{i=1}^{k}\eta_{i}$$ (8)

$$\mu (k)=\sum_{i=1}^{k}i\;\eta_{i}$$ (9)

### Validation and calculation of water inundated areas

The validation of the classified map was done through calculating a confusion matrix. The Sentinel -1 SAR classified images of 9^th^ and 21^st^ August 2018 were validated utilizing corresponding Sentinel-2 optical images, acquired on the 10^th^ and 20^th^ August 2018. The optical Sentinel 2 image acquired on 10^th^ and 20^th^ August were mostly affected with cloud cover therefore, these images were first preprocessed to remove clouds/ bad pixels. The cloud masking utilizes Sentinel-2 Band QA60, a quality flag band, to identify and mask out flagged cloud and cirrus pixels. After that, reference samples were randomly identified and collected using visual interpretation approach, for water and non-water class for the area that were not masked out due to cloud cover. The areas which were masked out due to cloud cover in sentinel-2 data, the reference samples were collected using sentinel-1 data.

**Fig 3:**
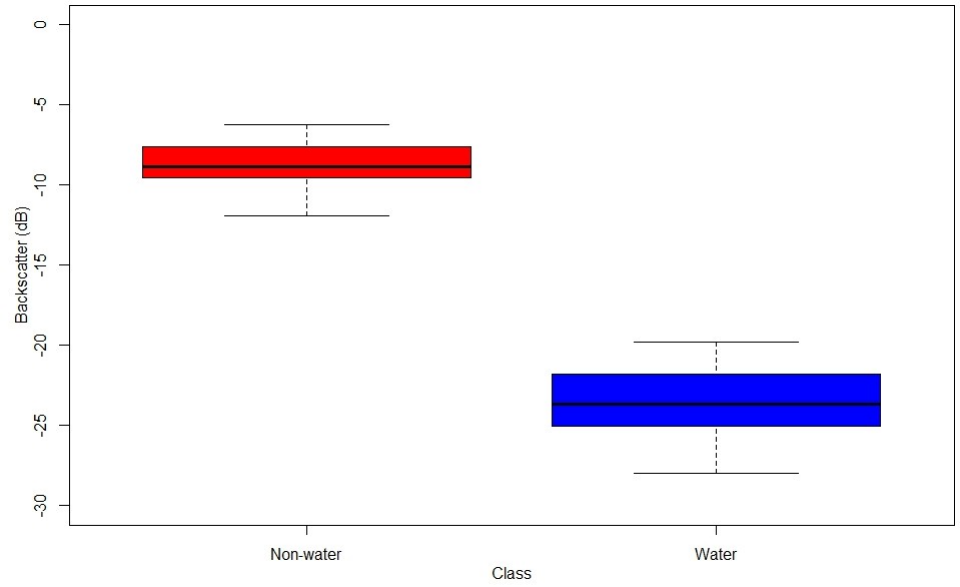
Backscatters values of non-water and water class.

The collected reference samples (from sentinel-2) were overlaid on Sentinel-1 SAR images of 9^th^ and 21^st^ August 2018. The backscatter values from water and non-water class were extracted and examined. It was observed that water class (Backscatter value = -23.53±2.44 db) exhibits low backscatter than non-water class (Backscatter value = -8.78±1.49 db) as shown in Fig 3. Then the same rule was utilized for collecting reference samples from Sentinel -1 SAR data (random sampling) for the areas which were affected by clouds. The aptness of the Otsu’s method was assessed by deriving not only the overall accuracy, but also individual class accuracies. The validation was also performed by (i) comparing the area submerged under water in the classified images of flooded (August 2018), non-flooded (January 2018) and previous year’s same month data (August of 2015, 2016 and 2017); and (ii) analyzing the rainfall data for confirming the flood event.

### Analyzing the rainfall data

Climate Hazards Group Infra-Red Precipitation with Station data (CHIRPS) is a quasi-global rainfall dataset prepared by blending satellite imagery with in-situ station data [55]. It has daily temporal resolution with 0.05 degree spatial resolution. Studies has been done in the past to evaluate the accuracy of the CHIRPS data over Indian region. Gupta, V. et al., [56] reported higher correlation with station data (>0.80) and lower systematic error (<10%) of CHIRPS rainfall over Kerala region. Similar results were also observed by Prakash, S. et al., [57]. This signifies that the CHIRPS rainfall has good potential for hydro metrological studies in this region. The linear trend analysis was performed to study the overall rainfall trend over the study area. Cumulative rainfall plot is used to analyze the variation of precipitation during monsoon season June, July and August (JJA) between 2015 and 2018. This period was selected to comply with the inundation analysis. Trend analysis during JJA (1981–2018) was also performed to monitor the long term rainfall pattern. The selected period of rainfall data was based on the recommendation of World Meteorological Organization (WMO)—WMO recommends a period of at least 30 years for the assessment of climatic change [58]. The advantage of having climatic data for longer period enables us to perform comparison of trends across different flood effected locations.

## Results and discussion

**Fig 4:**
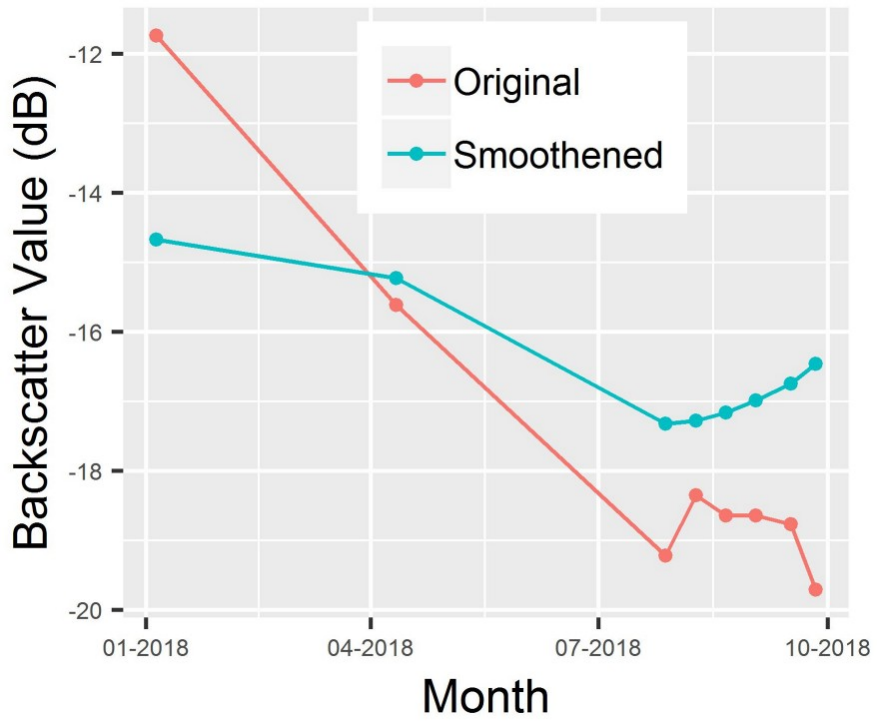
Time series backscatter signal at flooded location.

Time series backscatter profile was also examined at the flooded location as shown in Fig 4. The backscattered vs. time curve was smoothed utilizing Fourier transform smoothening technique [59]. Since water behaves as a smooth surface, the graph shows sudden decrease in the backscatter signal at the time of the flood (August 2018). Changes in the backscatter value describe the occurrence of the flood event in August 2018. The August 2018 flood event was also reported in the news [60,61]. The low backscatter values (function of surface roughness) in the month of August 2018 were due to the increase in water inundation (mainly due to the flood event) which again went up in September 2018 because of the decrease in water inundation (post flood event).

**Fig 5:**
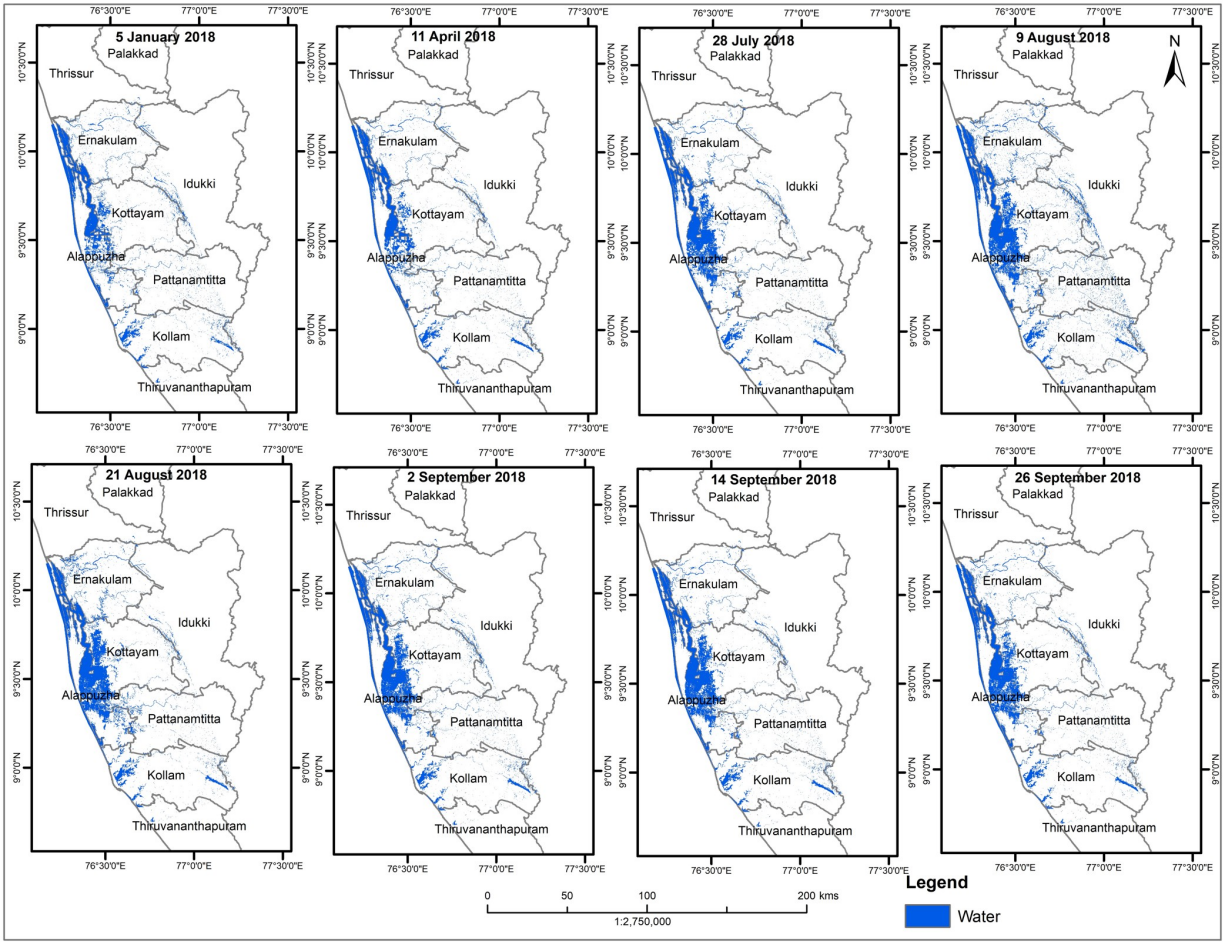
Water inundation map from Jan.–Sept. 2018. Shapefile reprinted from GADM database under a CC BY license, with permission from Global Administrative Areas (www.gadm.org), original copyright 2018. The figure was made with ArcGIS 10.3 under a CC BY license, with permission from ESRI (www.esri.com).

**Fig 6:**
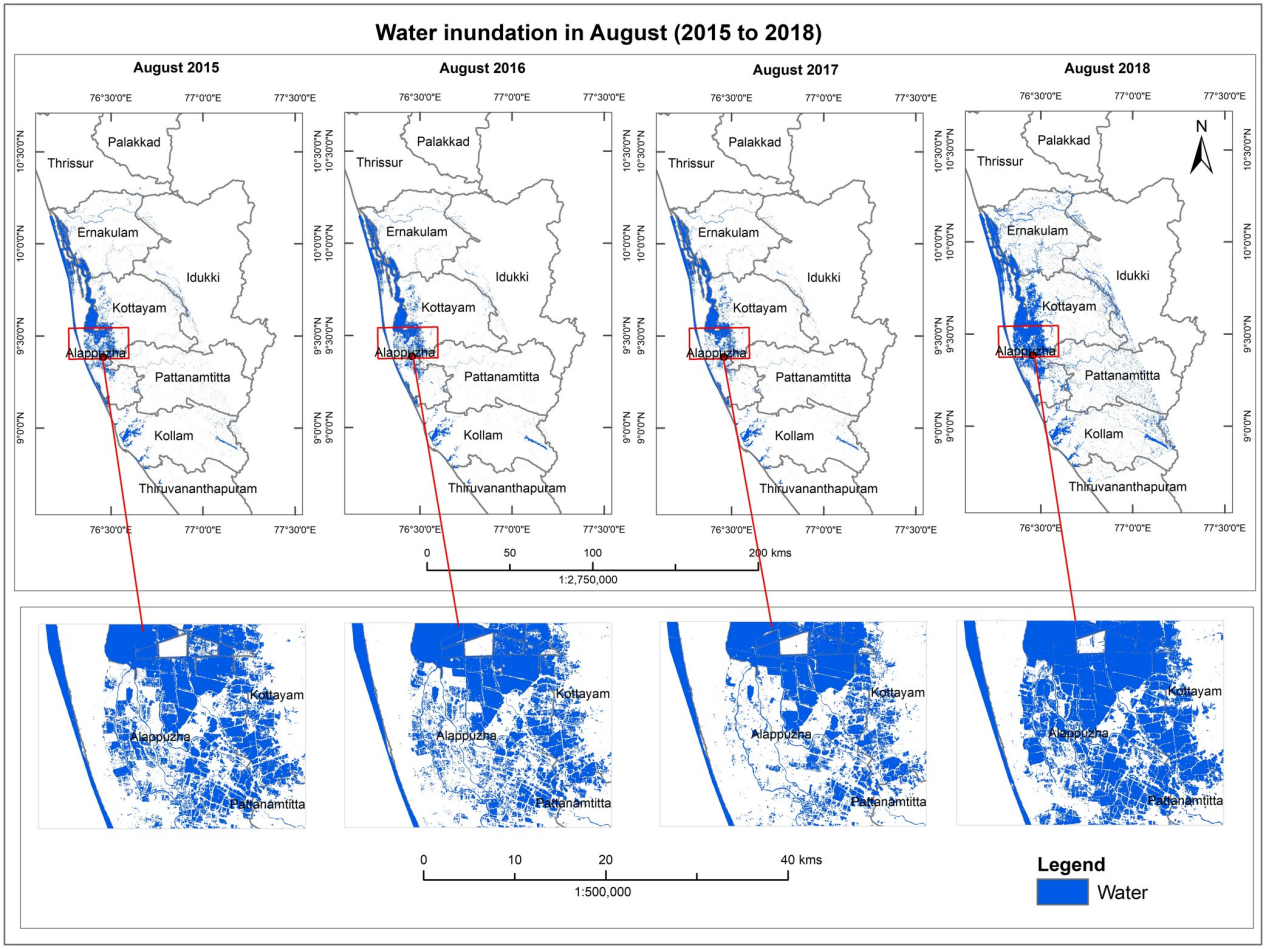
Water inundation map from Aug. (2015–2018). Shapefile reprinted from GADM database under a CC BY license, with permission from Global Administrative Areas (www.gadm.org), original copyright 2018. The figure was made with ArcGIS 10.3 under a CC BY license, with permission from ESRI (www.esri.com).

Water inundation maps from 2015–2018 and different seasons of 2018 were developed as depicted in Figs 5 and 6 respectively. Our analysis clearly show that the area submerged under water increased in the classified map of August 2018 as compared to the maps of the same month from 2015 to 2017 (Table 2). Similarly, in the classified images, water covered areas observed were less in pre-flood and post flood acquired data of 2018 (January-April and September) (Fig 6 and Table 2).

**Table 2:** Water inundated area observed in different dates data.

Event	Date	Area (Hectares)	Year
Before Flood	Aug. 13	88540.11	2015
	Aug.17	84210.56	2016
	Aug.14	80516.85	2017
	Jan. 5	66955.46	2018
	April 11	70923.45	
Starting of Flood	July 28	92463.39	
During Flood	Aug.9	109147.67	
	Aug. 21	105967.76	
After Flood	Sept. 2	95617.21	
	Sept.14	93871.25	
	Sept 26	90695.02	

**Fig 7:**
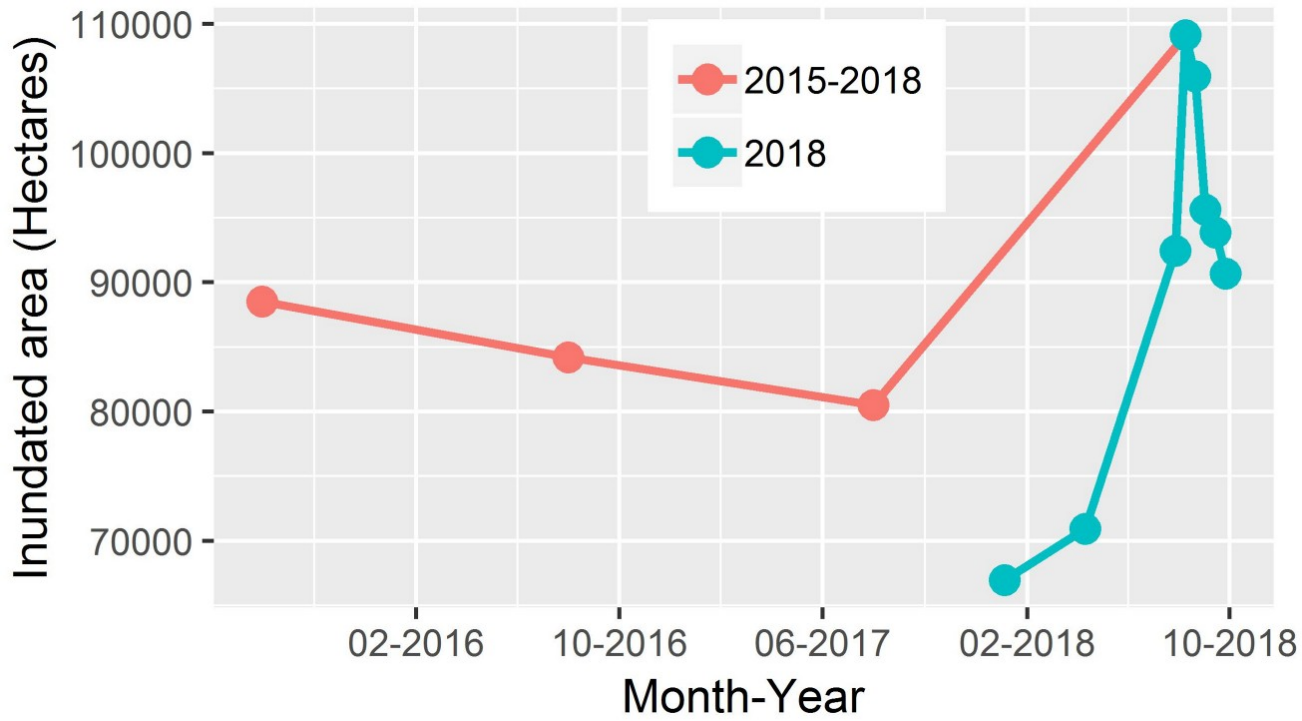
Water inundation area from Aug. 2015- Sept. 2018.

Fig 7 shows the graph between the different years (August 2015–2018) and the area submerged under water; and Fig 5 depicts a graph between different seasons (2018) and the area covered by water. It can be interpreted from the above graphs that the extent of the area in August 2018 is maximum as compared to other years (2015–2017) and seasons (January-September 2018). This clearly indicates the occurrence of a flood event in August 2018.

**Fig 8:**
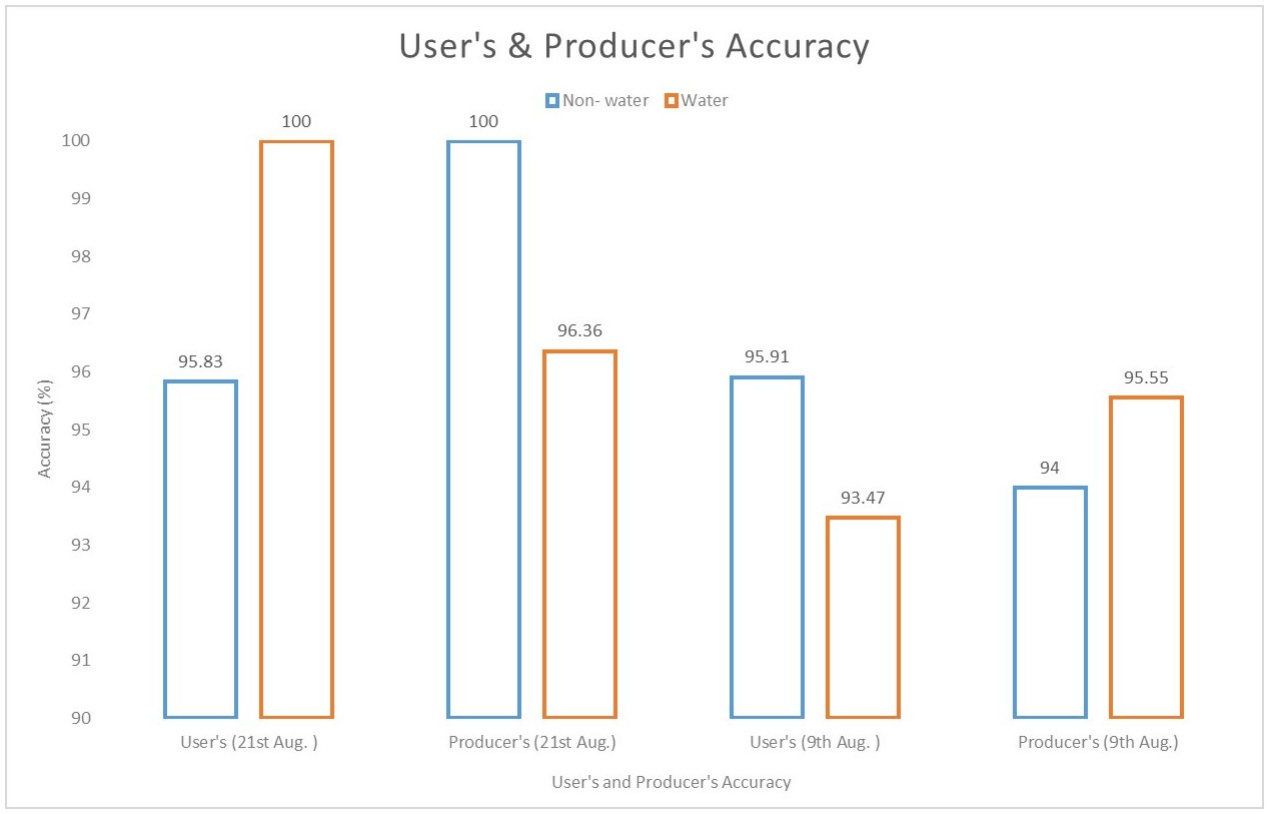
User’s and producer’s accuracy.

The accuracy assessment was performed on the 9^th^ and 21^st^ August 2018 flood maps. The overall accuracy of the classification was found to be 94.73% and 94.71% with a kappa coefficient of 0.87 and 0.88 respectively. The individual class accuracy of the classification is depicted in Fig 8. High classification accuracy demonstrates the potential of the Otsu’s algorithm for classifying the water and non-water pixels.

Sentinel-1 SAR operates in C band with its penetration capability ranges between ~5.6cm. Presence of clouds and rain droplets with thickness more than 7.5 cm at the time of data acquisition attenuates signal which result in noisy sentinel-1 images. This may be the reason for misclassification. However, using L or X band sensors can overcome this kind of uncertainty as these sensors have high penetration capability then C band.

**Fig 9:**
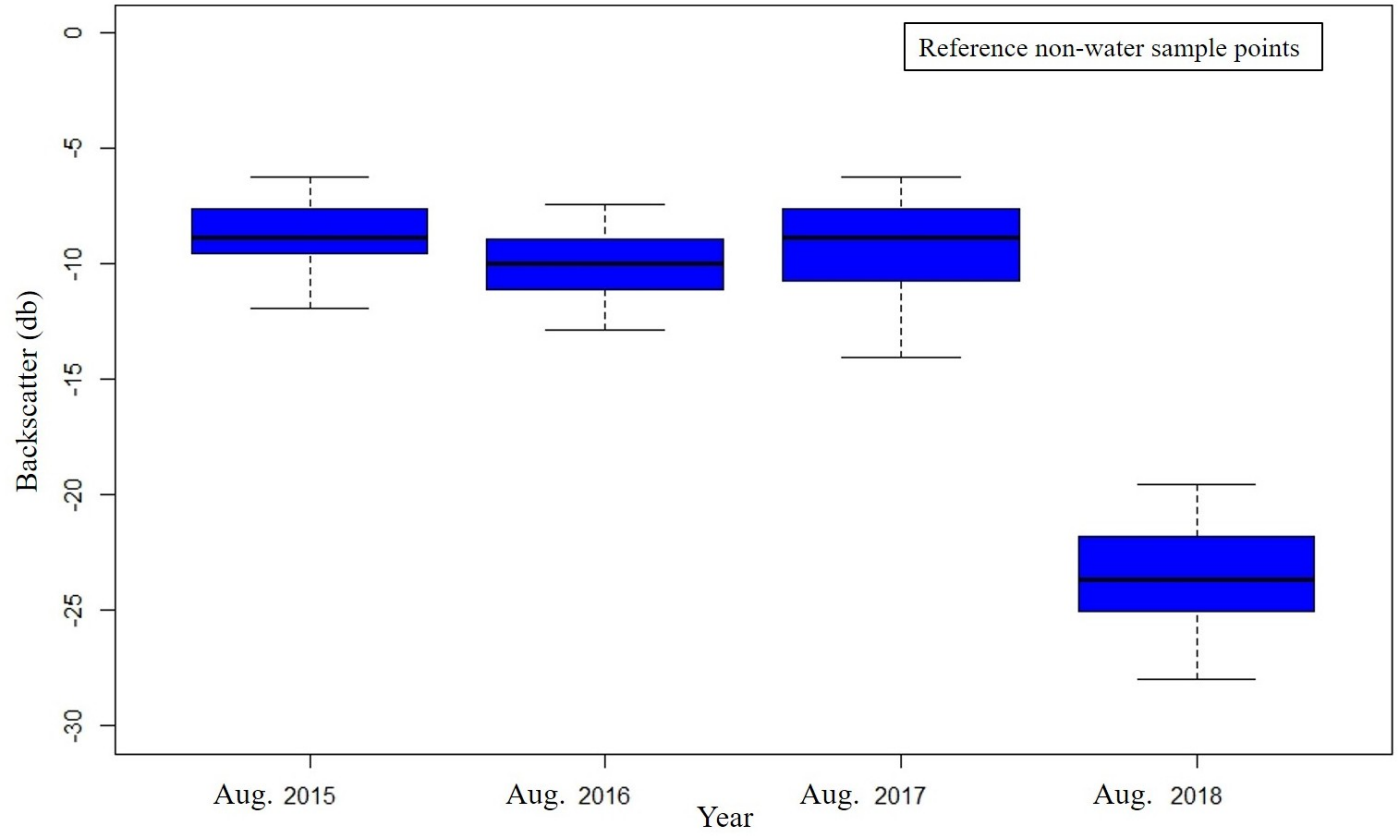
Backscatter values of non-water sample points from Aug. 2015–2018.

**Fig 10:**
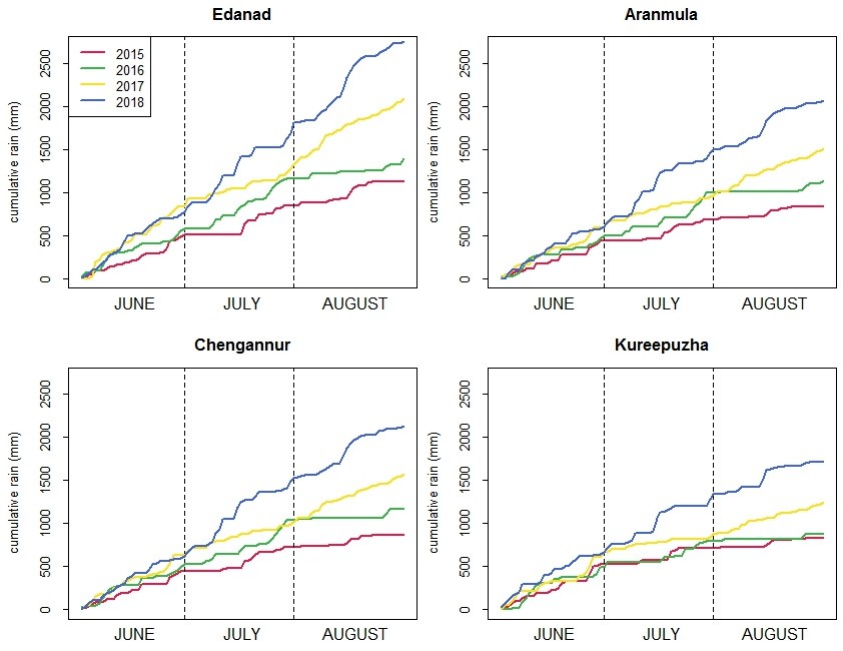
Cumulative rainfall for June July Aug. from 2015–2018.

The variation of the backscatter values was also examined using non-water sample points used in the validation. The backscatter response of the non-water sample points were examined for the month of Aug. 2015–2018 as depicted in Fig 9. It was observed that during the flood event (Aug. 2018), the backscatter values of the non-water class exhibits low backscatter values with a mean and average deviation value of -23.52 ± 2.14 db respectively. Contradictory, the backscatter values of the non-water class in non-flood year’s shows low backscattering i.e. -7.45 db ≤ Mean backscatter≤ -6.23 db and average deviation of ±1.8 db respectively. In addition to inundation delineation of the 2018 flood event, cumulative rainfall for the Kerala district was analyzed to provide further context to the magnitude of the flooding event. Fig 10 shows the cumulative rainfall during JJA for four different locations which were severely affected by floods. It was observed that all the locations received more amount of rainfall during 2018 as compared to other years (2015–2018).

Cumulative rainfall during 2018 was calculated to be 1.5 times higher than the average of cumulative rainfall for the period 2015–2018. This clearly signifies that rainfall was one of the driving factors for the flood event. Hunt et al., [31] have done the reservoirs capacity assessment of the six major reservoir of Kerala using WRF hydrological model where they depict that flooding event would have not taken place if the capacity of the six reservoirs would have been 34% more.

**Fig 11:**
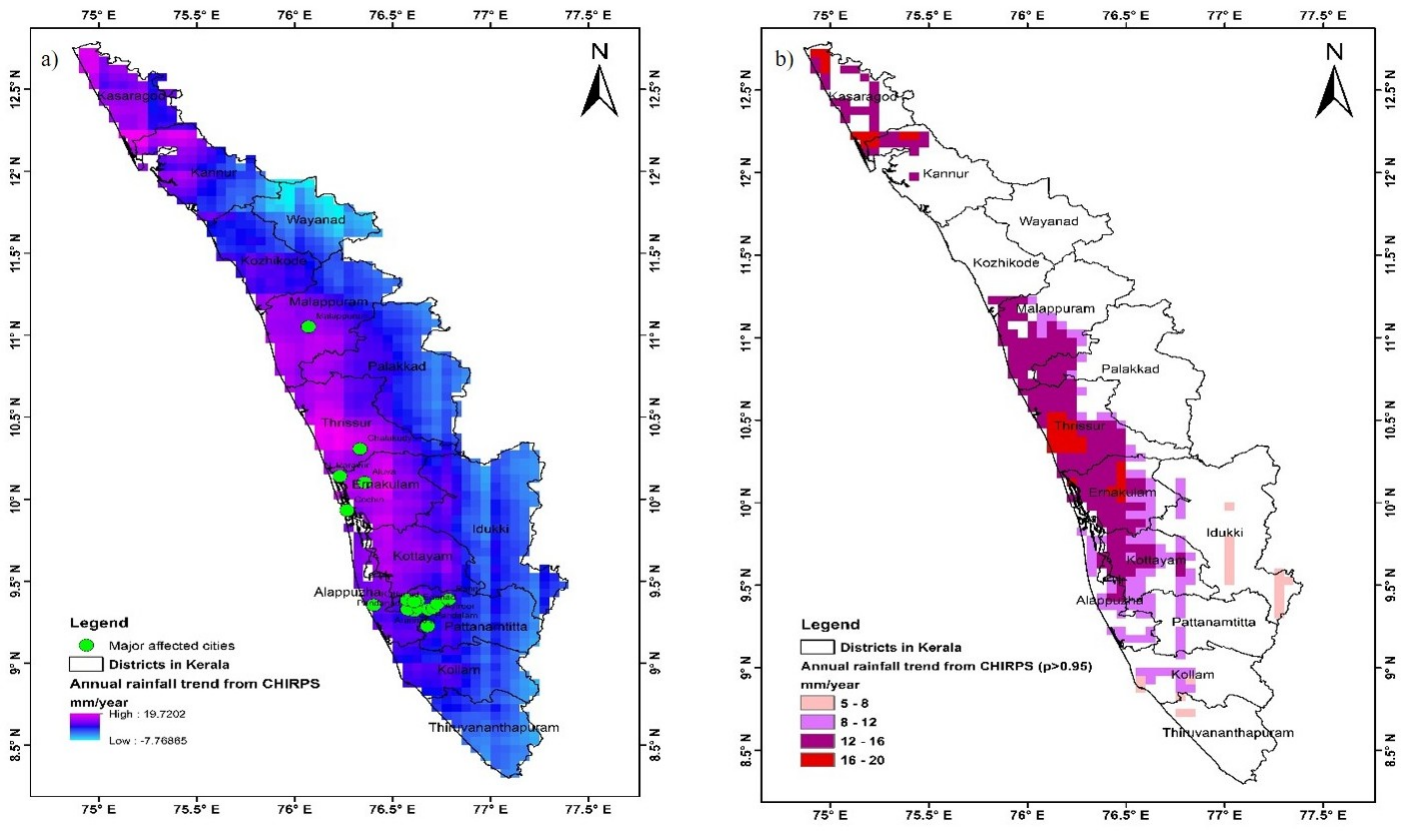
a) Pixel wise trend analysis of annual monsoon rainfall from 1981–2018 over Kerala, India; b) Pixel wise signification trend analysis (95% confidence level.) of annual monsoon rainfall from 1981–2018 over Kerala, India. Shapefile reprinted from GADM database under a CC BY license, with permission from Global Administrative Areas (www.gadm.org), original copyright 2018. The figure was made with ArcGIS 10.3 under a CC BY license, with permission from ESRI (www.esri.com).

Further analysis was performed to detect long term (1981–2018) rainfall trend during JJA. Fig 11a depicts the spatial variability of annual monsoon trend of rainfall over Kerala state from 1981–2018. It was observed that majority of the locations in Kerala have shown positive trends. Statistically significant trend (confidence level >95%) was extracted and illustrated in Fig 11b. It was observed that major flood affected locations (described in methods and material section) in Kerala have shown statistically significant increasing trend of JJA rainfall from 1981–2018. The increasing trend varies between 5 to 20 mm per year. Similar findings were depicted in Ali et al., [32] and Sharmila S, [62] where surface hydrological model was utilized to observed and projected changes in extreme precipitation and flood events. Their study demonstrate that the multi-day flood events are projected to increase with a faster rate in the future than the single day events.

## Conclusions and future recommendations

Swift and accurate estimation of water inundated areas can provide valuable and timely information during flood events. In this study, we assessed the Otsu thresolding approach using Sentinel-1 SAR for mapping flood areas. The analyses were done on the GEE platform. The conclusions that can be drawn from this study are as follows: (i) currently available Sentinel-1 SAR allows data retrieval in all weather conditions therefore, has a good potential for mapping water inundated areas at the time of floods specifically in Kerala where more flood are likely to occur in near future (Fig 11a & 11b); (ii) Otsu’s method is capable of automatically detecting thresholds for separating water and non-water pixels without requiring user dependent parameter (reference points from the ground) unlike supervised machine learning methods; and (iii) the GEE platform can be used for processing and analysis of the inundation maps without the need for downloading data or high computing hardware. The automated and on-the-fly performance at acceptable accuracy level proves the utility and potential of GEE applications for rapid response in flood inundation mapping. While this paper highlights a proof of concept, further analysis is needed to explore the VH Polarization and band ratio techniques such as VV/VH. Since the Otsu method has been implemented and tested in GEE environment, the method can be tried on other areas for mapping real time flood events without requirement of ground sample points, except for validation. Though Otsu have shown good performance with far and wide case studies for flood mapping. It has limitations, Otsu assumed to have bimodal histogram distribution and also assumed to possess a deep and sharp valley between two peaks. In the study area if the object area is small compared with the background area, the histogram no longer exhibits bimodality. Therefore, Two-dimensional Otsu’s method can be consider as it performs better image segmentation in noisy images [63]. Additionally, how information can be utilized and shared through social media could be explored for further validation and dissemination of the findings for rapid response as described in Babu et al., [64].

It is important to note that the natural occurrence and impact of floods can be magnified by socio-economic development, including encroachment on the floodplains and inadequate drainage management [65]. In addition, traditional knowledge of dealing with floods has been forgotten, which have the potential to offer site-specific knowledge and critical guidance to planners and decision makers. Flooding causes economic and livelihood losses especially in developing countries where low-income earners undergo great stress. Losses due to floods reduces the asset base of households, communities and societies through the destruction of standing crops, dwellings, infrastructure, machinery and buildings, in addition to tragic loss of life. Despite the rich natural resources and water, people living in these areas are poorer as compared to the rest of the country, and this is primarily due to the annual floods and related devastation. The resulting financial burden is often back-breaking for the communities. The pressure to maintain the day to day expenses, education of children, rituals, health amongst these disasters, increasing poverty, and few available adaptive mechanisms has sometimes led to migration from such areas. Flood and migration have direct relation as higher the flood, higher is the migration rate. It is important to note that floods impact different sections of society differentially, and requires a thorough understanding of flood-related issues. Floods have adverse impacts such as the risk to life and property in the coming years, both because floods are likely to be more frequent, and because increase in population is likely to result in more people settling in areas vulnerable to flooding. An increase in floods is likely to influence prevalence of water-related diseases and contaminate water sources whereas, the insufficient water for hygiene purposes during dry periods is likely to increase the risk of water-washed diseases. Given the projected changes in precipitation, it is likely that there is going to be serious costs for communities and sectors here. In view of the above, there is an urgent need for actions to assist communities and dependent sectors to adapt. Therefore, the various heavily water-dependent sectors—agriculture, industry, energy, navigation and water supply and sanitation—need to cooperate to work towards achieving a resilient State.

## Supporting information

S1 File (7Z)
